# Risk Factors for Positive Resection Margins Following Breast-Conserving Surgery

**DOI:** 10.7759/cureus.76406

**Published:** 2024-12-26

**Authors:** Gabriella Truda, Sarah Howells, Madeleine Berry, Nour Al-Shurbasi

**Affiliations:** 1 Department of Breast, Plastic and Reconstructive Surgery, Royal Hallamshire Hospital, Sheffield, GBR; 2 Department of Breast Screening and Breast Imaging, Royal Hallamshire Hospital, Sheffield, GBR

**Keywords:** breast-cancer, breast cancer pathology, breast-conserving surgery, ductal carcinoma in situ (dcis), margin re-excision, non palpable breast cancer, positive margins, resection margins, wle - wide local excision

## Abstract

Background

The incidence of margin re-excision following breast conserving surgery (BCS) is a quality measure in the National Health Service. The threshold is less than 20% of all BCS procedures. Despite three decades of studies and a wealth of literature identifying multiple factors associated with increased risk for margin involvement, an accepted threshold rate affecting one in five procedures remains high.

Aim

The aim of the study was to identify adverse features that continue to compromise successful margin clearance despite the recognition of risk factors and the implementation of strategies designed to minimise those risks.

Methods

All margin re-excisions following BCS for invasive breast carcinoma and ductal carcinoma in situ (DCIS) performed from October 2013 to September 2018 were retrieved from the database of a single institution. A total of 1379 patients underwent BCS during the period considered, 194 of which needed margin re-excision. Radiological investigations and histopathology reports for each patient were retrieved. Lesion size and focality on mammogram, ultrasound (US) scan, and magnetic resonance imaging (MRI), and histopathologic tumour characteristics were recorded and analysed.

Results

The overall re-excision rate was 14.06% (194/1379 patients). Margin re-excisions cleared 69% (134/194) of wide local excision cavities that had at least one involved margin. 53% (103/194) of patients had no further disease after one attempt at re-excision and 15.9% (31/of 194) had further disease, which was cleared after re-excision. Another 15.9% (31/194) had disease within the shave with involved margins. In this sub-group the presence of DCIS at the new resection margin accounted for 90.3% (28/31) of cases, 3% (1/31) were invasive ductal carcinoma (IDC) and 6% (2/31) were unrecorded. In the sub-group of patients who had an excised margin with pathology and a new clear margin (15.9% of all re-excisions), DCIS was found in 61% (19/31) of cases, IDC in 12.9% (4/31), invasive lobular carcinoma (ILC) in 6% (2/31) of cases, lobular neoplasia (LN) in 12.9% (4/31), mixed IDC and DCIS in 6% (2/31)of cases. The correlation between imaging size and actual histopathological size has shown a statistically significant discrepancy in this cohort. The median size on histology was 22 mm, compared to a median size of 16 mm on mammography, 14 mm on ultrasound, and 17 mm on MRI.

Conclusion

According to our cohort of patients, the most consistent factor associated with a re-excision was the presence of DCIS at the resection margin, whether pure DCIS or IDC admixed with DCIS. The comparison between tumour size on imaging and final histopathological size revealed the best correlation with mammogram followed by US. The weakest correlation was with MRI.

## Introduction

Breast cancer is the most common cancer in the UK, with around 55,000 new cases diagnosed annually [1]. The primary treatment is surgery, most commonly breast-conserving surgery (BCS), which gives comparative outcomes to mastectomy when combined with radiotherapy and when adequate resection is achieved.

The guidance of the National Institute for Health and Care Excellence (NICE) is followed in most breast units in the UK. The recommended margin was 1 mm for invasive carcinoma and a 2 mm for ductal carcinoma in situ (DCIS) prior to 2018 . This was updated in 2018 to "no ink on tumour" for both invasive cancer and ductal carcinoma in situ (DCIS) [2]. The 2018 NICE guidelines were in line with the 2014 guidelines of the Society of Surgical Oncology (SSO) and the American Society of Therapeutic Radiology and Oncology (ASTRO) [3,4].

Re-excision following BCS is psychologically stressful for patients and sometimes leads to poor cosmesis. Furthermore, it impacts negatively on national waiting time targets for cancer treatment and represents an additional cost for the National Health System (NHS) of the UK.

The adoption of the latest guidelines can potentially decrease unnecessary re-excisions, thus reducing delays in adjuvant treatment and alleviating pressure on NHS [5,6]. This would also lower patient anxiety due to re-operation and improve cosmetic outcomes, resulting in better patient satisfaction.

Factors identified in the literature that impact re-excision rates include screen-detected impalpable lesions, lack of preoperative diagnosis, neoadjuvant chemotherapy, multi-focal lesions, presence of in situ component on pathology, and surgeon experience [7].

## Materials and methods

All patients who underwent re-excision surgery following BCS for invasive breast carcinoma or DCIS at Sheffield Teaching Hospitals Breast Unit from October 2013 to September 2018 were retrospectively reviewed via the surgical database (ORMIS - Operating Room Management Information System). A total of 194 patients were identified out of 1379 who underwent BCS.

We included in the study all patients diagnosed with invasive breast carcinoma or DCIS who underwent BCS and had at least one involved or close margin according to the pre-2018 NICE guidelines (1 mm margin for invasive carcinoma and 2 mm margin for ductal carcinoma in situ).

Sarcomas were excluded from the count of wide local excisions performed in the given period, along with all benign cases, including phyllodes tumours and a leiomyoma.

For each patient, demographics and imaging size on mammogram, ultrasound (US) scan, and MRI were recorded along with histopathology of lesion excised in the index surgery and subsequently in all re-resections to achieve clear margins. 

A specimen X-ray was performed intraoperatively, on-site, for all BCS cases, and re-resections were guided by palpation combined with specimen X-ray findings.

Margin re-excisions were categorized into three groups:

Group 1 had a resection with no lesions. Complete resection was achieved prior to margin re-excision surgery. The resection margin specimen showed only healthy breast tissue.

Group 2 had adequate resection with lesions. Margin re-excision showed pathology (invasive cancer and/or DCIS) with adequate healthy tissue around it.

Group 3 had inadequate resection with lesions. Re-excision specimen showed pathology with close or involved new margin which ended in another margin re-excision or mastectomy.

Statistical analyses were performed using SPSS software (IBM Corp., Armonk, USA). Differences between groups were determined using the Chi-squared test, Friedman test, and individual Wilcoxon signed-rank test with Bonferroni correction applied.

## Results

Patients, disease, and surgical demographics

A total of 1379 patients, all females, with a median age of 60 years (age range 34.1-84.2), had BCS for invasive carcinoma or DCIS in our centre from Oct 2013 to September 2018, 194 of those had close or involved margin.

Re-operation rate

The global re-operation rate was 14.06% (194/1379 patients). 4.1% of total re-excisions ended with a mastectomy (8/194). The disease was present in 31.9% of re-excision specimens and in 100% of completion mastectomy specimens.

Imaging size (mammogram+US scan) vs. histological size

Our sample consisted of 66 patients who underwent further excision following the removal of a single breast tumour and whose imaging, with both mammogram and US scan, reported a smaller tumour size than that found on the histological specimen. The median size of the tumour on histology was 22 mm, the median size suggested by the mammogram was 16 mm, and the median size suggested by the US scan was 14 mm. The interquartile range (IQR) was 11.5 mm (26.50 mm - 15 mm) for histology, 12 mm (24 mm - 12 mm) for mammogram, and 10.5 mm (18 mm - 7.5 mm) for US scan.

A Friedman test was carried out as a non-parametric test for continuous, matched data. The Friedman test suggested a statistically significant difference between mammogram, US scan, and histological measurement (Chi-square 40.678, p = 0.000).

Further analysis was carried out using individual Wilcoxon signed-rank tests with a Bonferroni correction applied, giving a significance level of p < 0.017. These showed a statistically significant difference between histological and mammographic measurements (Z = -3.137, p = 0.002), between histological and ultrasound measurements (Z = -5.524, p = 0.000), and also between mammographic and ultrasound measurements (Z = -5.065, p = 0.000).

Size on mammogram vs. histological size

Our sample consisted of 72 patients who had a single breast tumour removed and required further excision, and who had a mammographic tumour measurement taken pre-operatively that was smaller than the histological measurement.

The median tumour size was 23.5 mm on histology and 14.5 mm on mammography. The IQR was 16.5 mm (32.750 mm - 16.250 mm) for histology and 12 mm (21 mm - 9 mm) for mammography.

A Wilcoxon signed-rank test was performed, which showed a statistically significant difference between the tumour size as measured on mammography and the tumour size as measured histologically, with a significance level of p < 0.05 (Z = -7.171, p = 0.000).

Size on US scan vs. histological size

Our sample consisted of 86 patients who had a single breast tumour removed and had subsequent further excision, and who had pre-operative a tumour measurement taken using a US scan that was smaller than the histological measurement.

The median tumour size on histology was 23 mm and the median tumour size on the US scan was 12 mm. The IQR was 14.25 mm (30.25 mm - 16 mm) for histology and 10 mm (17 mm - 7 mm) for the US scan.

Wilcoxon signed-ranks test was carried out, which showed a statistically significant difference between measurements taken on ultrasound scan and histological measurements, with a significance level of p < 0.05 (Z = -7.965 and p = 0.000).

Size on MRI vs. histological size

Our sample consisted of 12 patients who had a single breast tumour removed and subsequently underwent further excision, and who had pre-operative tumour measurement taken via MRI that was smaller than the histological measurement.

The median tumour size on histology was 28 mm and the median tumour size on MRI was 17.5 mm. The IQR was 19.5 mm (42.75 mm - 23.25 mm) for histology and 10 mm (22.75 mm - 12.75 mm) for MRI.

Wilcoxon signed-rank test suggested a significant difference between MRI measurement and histological measurement, given a significance level of p < 0.05 (Z = -3.062, p = 0.002); however, the validity of these findings may be weakened by the small sample size.

**Figure 1:**
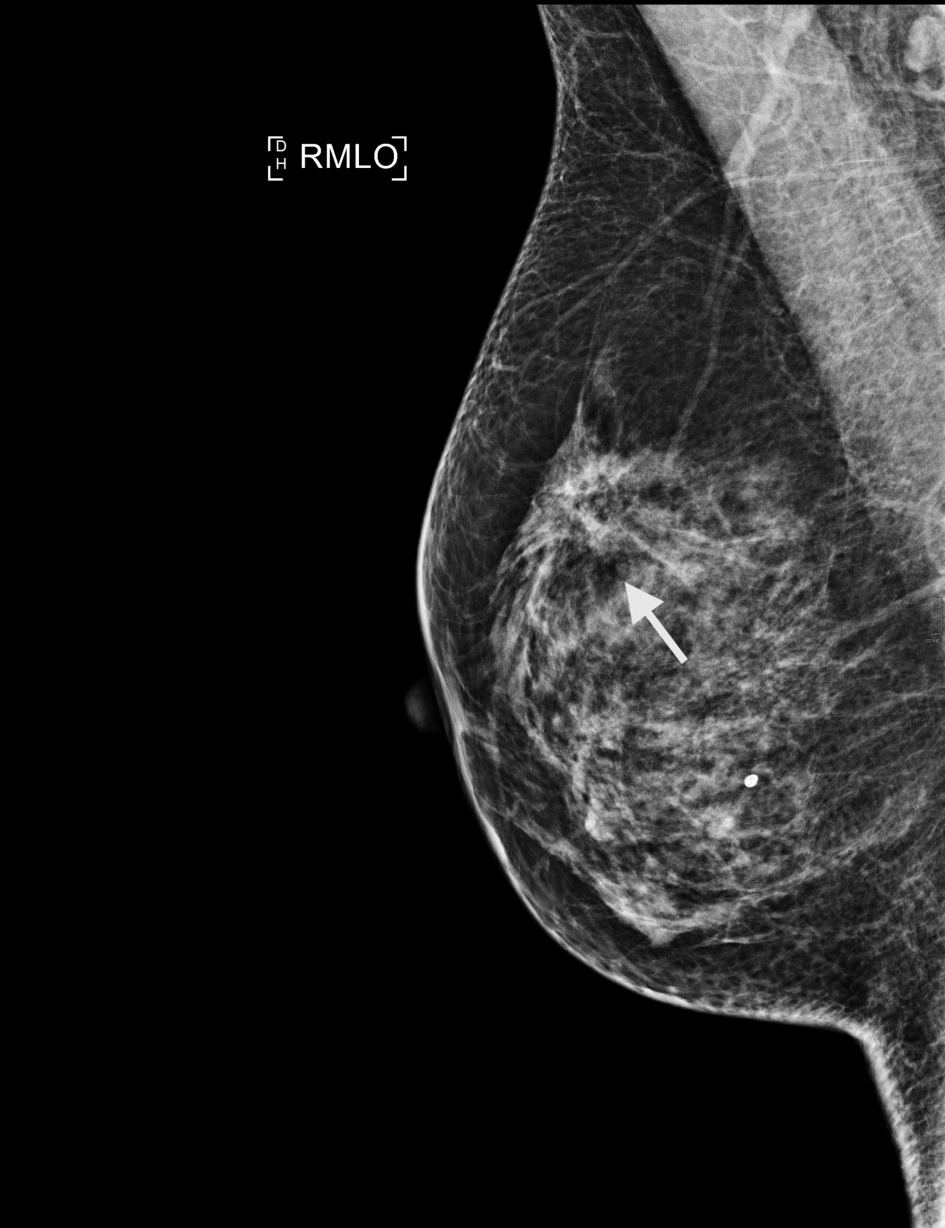
Case 1 - 49-year-old woman, right mammogram mediolateral oblique (MLO) view. The arrow indicates the stromal deformity at 12 o'clock position.

**Figure 2:**
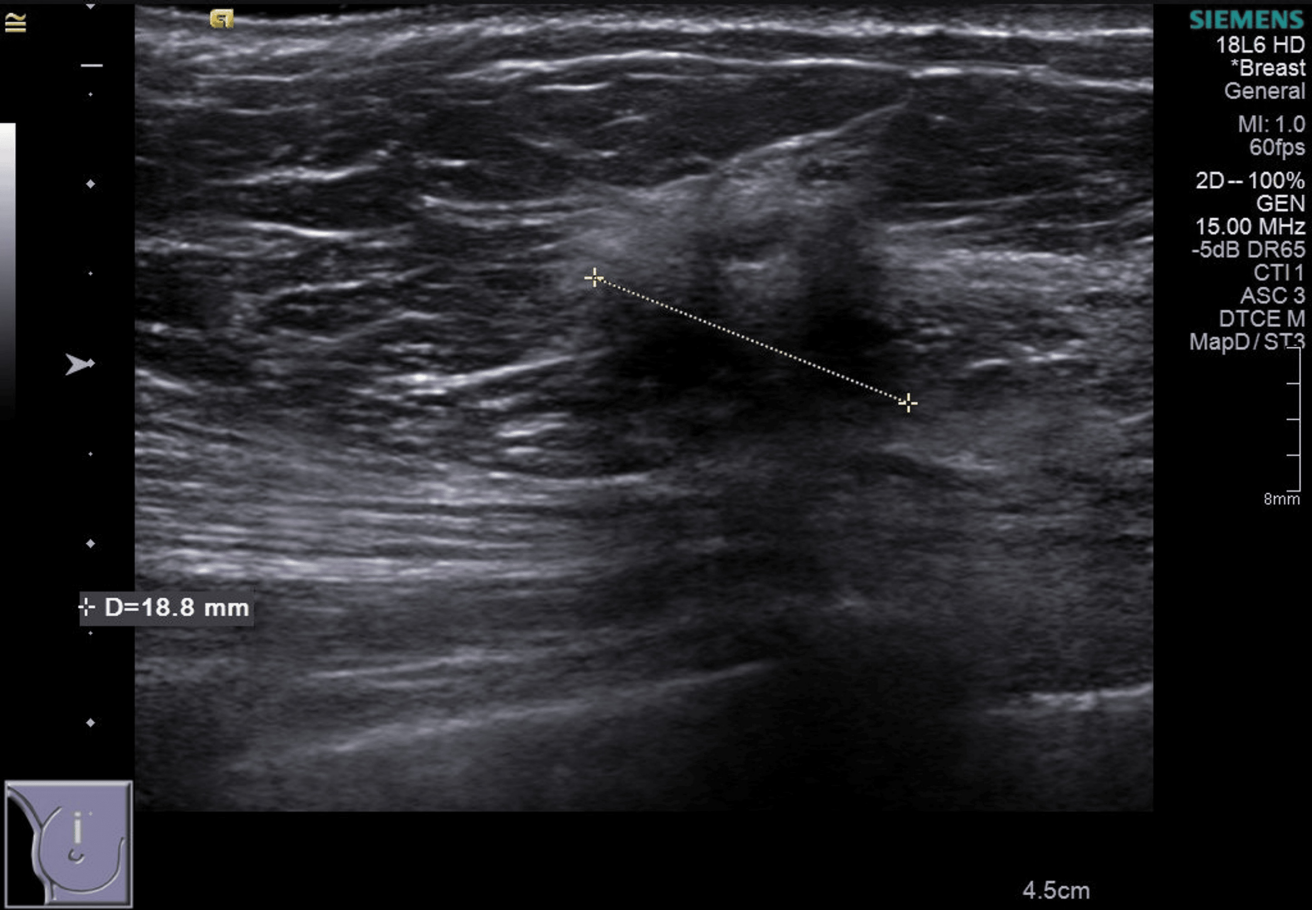
Case 1 - 49-year-old woman, targeted US scan of the right breast showing an 18.8 mm hypoechoic mass at 12 o’clock position.

Figures 1-2 show images of a mammogram and a US scan of a 49-year-old woman who needed a medial margin re-excision for a 49 mm invasive lobular carcinoma (ILC)+lobular neoplasia (LN) on histopathology (case 1). A bilateral mammogram demonstrated mixed-density breast tissue and a stromal deformity in the 12 o’clock position of the right breast, best seen on the right mediolateral oblique (MLO) view. The lesion was difficult to accurately measure but was at least 22 mm, M5. A US scan demonstrated an 18.8 mm hypoechoic, ill-defined mass at 12 o'clock, U5.

MRI on the same patient showed an 18 mm area of architectural distortion within the 12 o’clock position of the right breast, seen as a low signal on the T1 and T2 pre-contrast sequences. This corresponded to the biopsy-proven lobular carcinoma. There was very little enhancement of the lesion following contrast administration, and this reduced the reliability of the MRI in estimating the overall size of the disease accurately. This might explain why the MRI underestimated the size of the tumour seen on histopathology.

The limitations of MRI to accurately measure the size of a lesion and exclude further disease when a cancer does not demonstrate the usual early pathological enhancement patterns should be part of the multidisciplinary team (MDT) discussion so that the surgeon is aware of the potential for the tumour to be larger than the estimates given. 

Re-excision rate and histopathology of cancer

From October 2013 to September 2018, 1379 BCSs were performed in our institution, 194 of which had close or involved margins, equivalent to a re-excision rate of 14.06%. 69% of patients (134/194) were cleared following a first cavity shave, 53% of patients (103/194) had no further disease after one attempt at re-excision and 15.9% (31/of 194) had further disease, which was cleared by re-excision. Another 15.9% (31/194) had disease within the shave with involved margins. In this sub-group the presence of DCIS at the new resection margin accounted for 90.3% (28/31) of cases, 3% (1/31) were invasive ductal carcinoma (IDC) and 6% (2/31) were unrecorded. In the sub-group of patients who had an excised margin with pathology and a new clear margin (15.9% of all re-excisions), DCIS was found in 61% (19/31) of cases, IDC in 12.9% (4/31), ILC in 6% (2/31), LN in 12.9% (4/31), and mixed IDC and DCIS in 6% (2/31) of cases.

Table 1 shows the number of the different histopathological types of lesions found on specimens of first and second cavity shave. Three out of 194 patients had bilateral breast cancer and two of them required bilateral re-excision. Multifocality was present in 25.7% of cases (50/194). The analysis of the histopathological type of lesion in cases with inadequate or involved margins in this cohort has shown that mostly DCIS was present at the margin, whether solo or admixed with IDC (72.9%).

**Table 1:** Number of lesions on re-excision divided by histopathology per year Mixed: IDC + DCIS DCIS: ductal carcinoma in situ; IDC: invasive ductal carcinoma

Year	Invasive ductal	DCIS	Invasive lobular	Mixed	Lobular neoplasia	Total
2013	1	2	1	6	1	11
2014	4	5	1	14	0	24
2015	2	21	4	21	1	49
2016	5	8	6	24	3	46
2017	3	13	7	22	7	52
2018	9	7	1	10	1	28
Total	24	56	20	97	13	210

**Figure 3:**
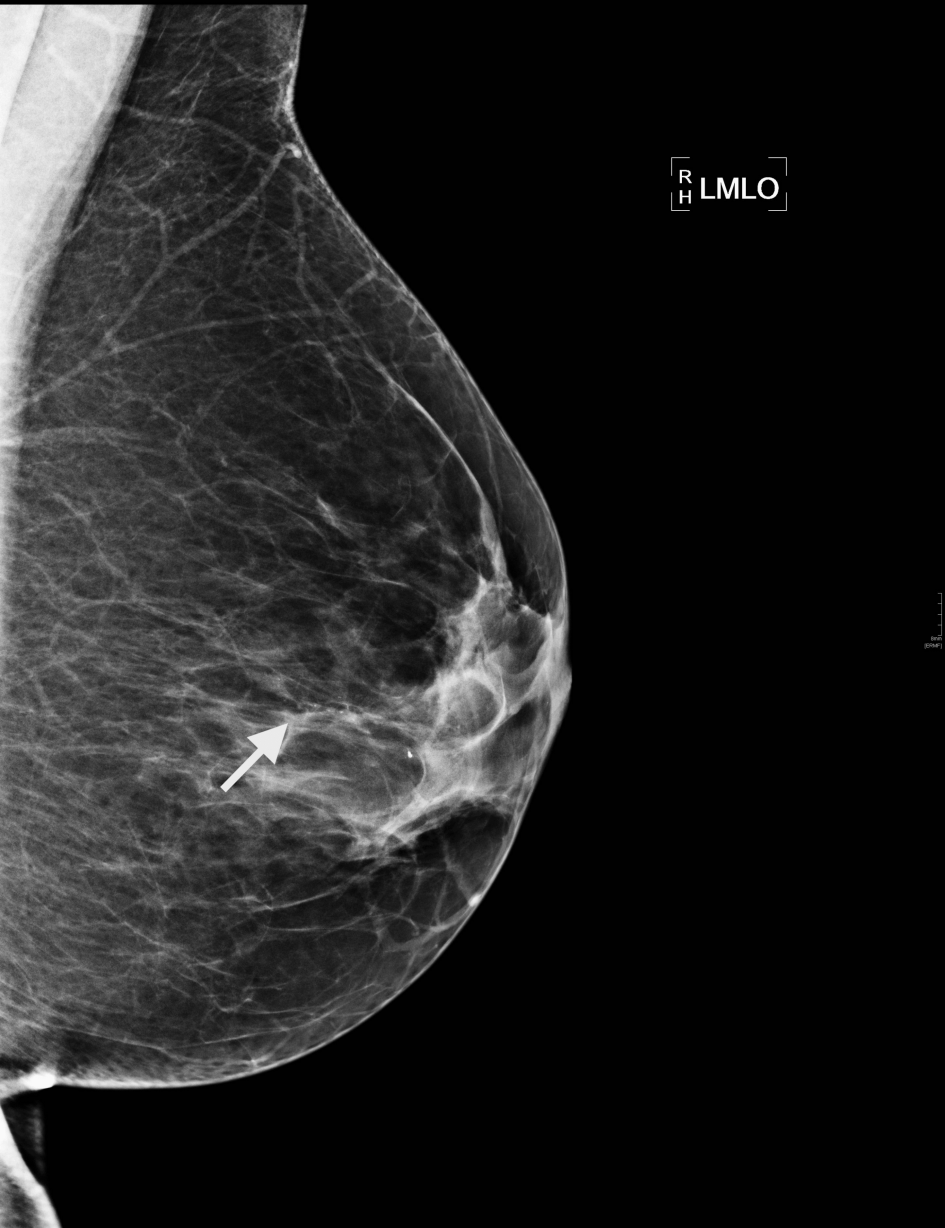
Case 2 - 46-year-old woman, left mammogram mediolateral oblique (MLO) view. The arrow indicates the linear micro calcifications.

**Figure 4:**
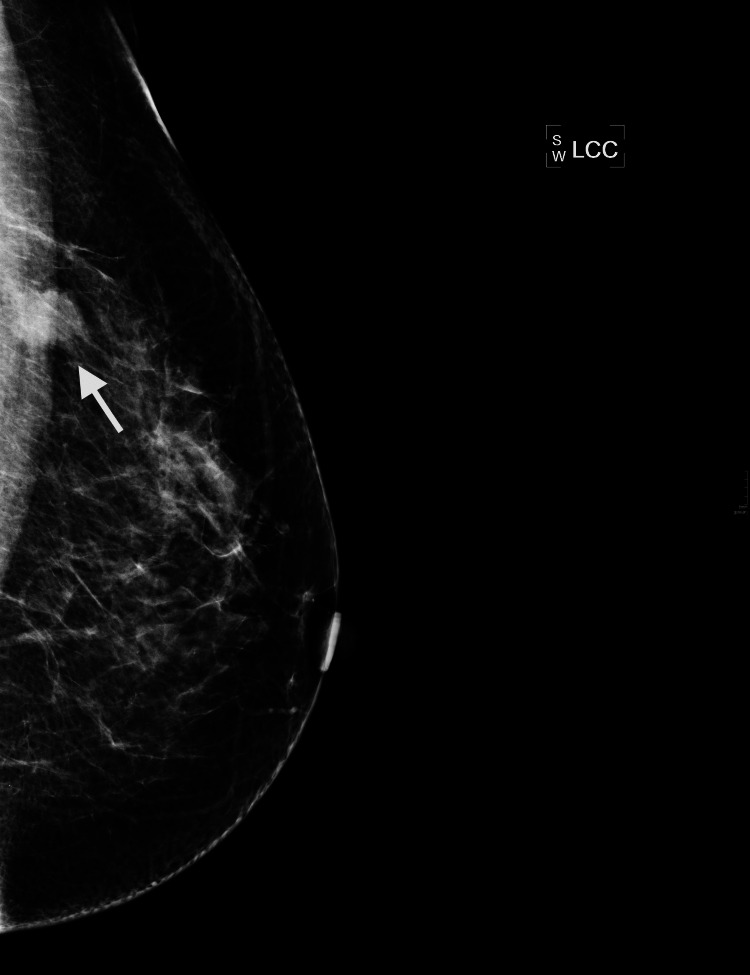
Case 3 - 57-year-old woman, left mammogram craniocaudal (CC) view. The arrow indicates the 14 mm mass.

**Figure 5:**
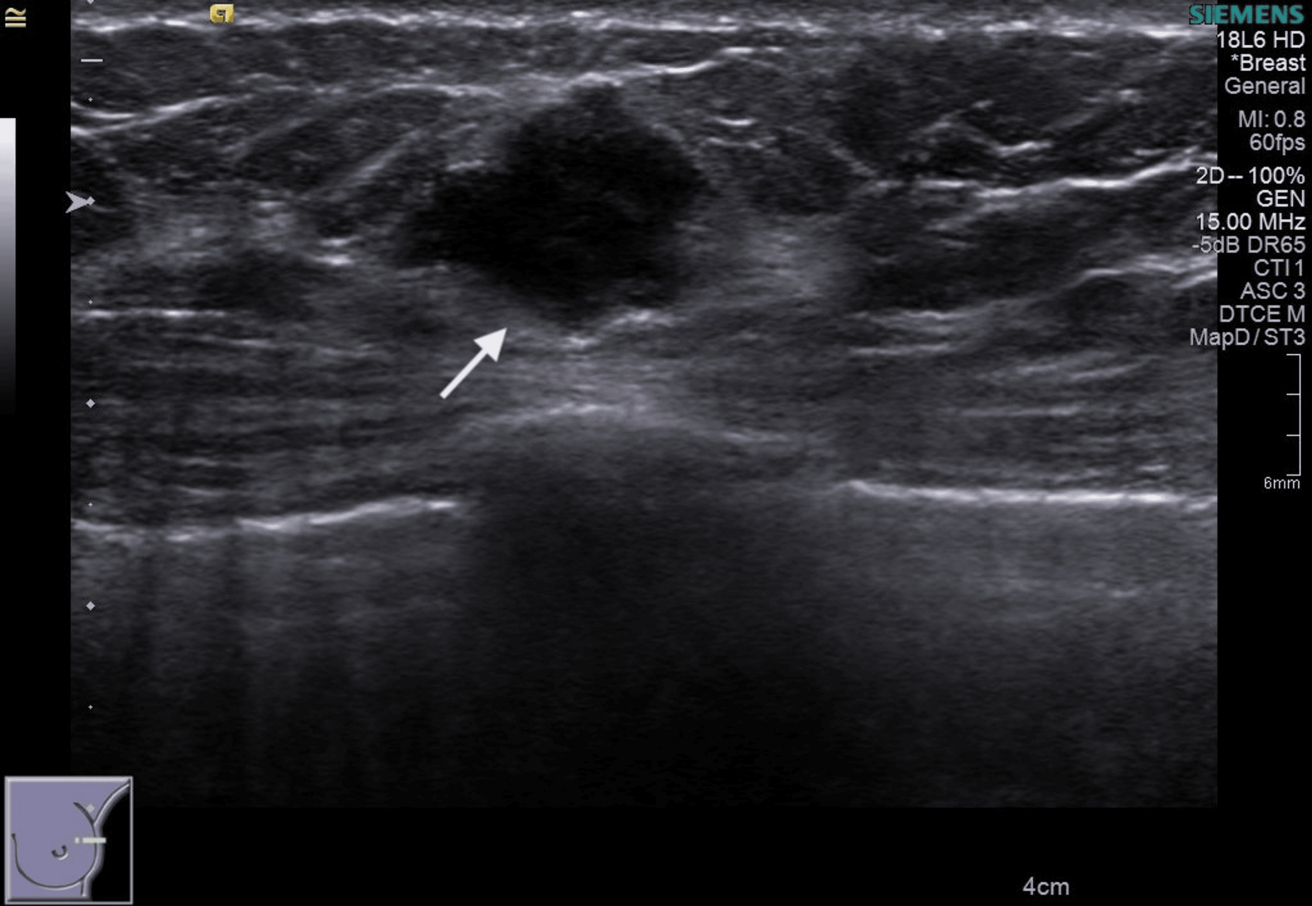
Case 3 - 57-year-old woman, left breast targeted ultrasound (US) scan of the upper pole. The arrow shows a 16 mm hypoechoic lesion.

Figures 3-5 show images of two cases (case 2 and case 3) that underwent a margin re-excision twice, and both ended with a mastectomy due to new positive margins. Case 2 was a 46-year-old woman with DCIS only on histology, and case 3 was a 57-year-old woman with IDC of non-special type plus high-grade DCIS.

Case 2 mammogram demonstrated 23 mm of new linear micro-calcifications within the left medial breast. As the US scan was normal, the patient had a stereo-guided biopsy with micro-calcifications within the specimen. 40-mm high-grade DCIS was present in the wide local excision and a 48-mm high-grade DCIS was found in the mastectomy specimen.

Case 3 was a screening recall for a 14 mm mass in the left upper breast on the mediolateral oblique (MLO) view, also seen on the extended craniocaudal (CC) view laterally, M5. A US scan showed a 16 mm hypoechoic mass, U5. No other lesion was identified on mammogram or US scan. The final histopathology report showed a 17 mm G3 IDC of non-special type with associated high-grade DCIS for a total extent of 25 mm.

## Discussion

Re-excision rates following BCS are variable between 0% and 40%, with a mean of approximately 20% in several large national databases [6,8].

This large retrospective cohort identified a reoperation rate following BCS of 14.06% at a tertiary referral breast cancer unit in the UK. Significant factors to predict a re-operation were the presence of DCIS at the resection margin and the discrepancy between imaging size and pathological size. 

DCIS at the resection margin is one of the recognised risk factors for re-excision, especially the non-calcifying variant, which is a radiological as well as a surgical challenge [7,9-12]. Whether in solo or associated with an invasive component, DCIS was identified as the most important factor in predicting close or involved margin in this cohort.

The discrepancy between imaging size and pathological size, already identified in the literature as a significant risk factor for re-excision, was confirmed in this cohort. The analysis of our data showed that MRI was the least accurate method in assessing the lesion size. The most accurate estimate was given by mammogram, followed by a US scan. Despite the due consideration that the accuracy of this result is weakened by the limited number of patients who had an MRI in this cohort, the observed discrepancy, whether over or underestimated, is in line with previous literature which reported a concordance rate of 56% between MRI size and pathological size [13]. 

Despite the large number of cases of BCS in this cohort, some limitations of this study have to be considered. The variability in the experience of consultant surgeons in the unit was significant, ranging from zero to 30 years. All patients were under a consultant’s name but some operations were performed by trainees; however, all trainees were directly supervised by consultants or senior fellows. In addition, surgeons with many years of experience were likely to be more precise in interpreting the X-ray specimen, and while some surgeons were more prone to taking cavity shaves in the index surgery, others tended to wait for the result of the final pathology report to avoid unnecessary re-resection. Also, the experience of the radiologists of the unit was variable, and this may have had an impact on the precision of wire insertion in the case of non-palpable lesions.

The debate on adequate margins is a long-standing one. Margin re-excision following BCS is generally a simple procedure, but it causes delays in adjuvant treatments and adversely affects the patient’s psychology and satisfaction, having an impact on cosmesis as well as on costs for the national health service. The consensus on adequate margin for oncological safety has changed with time while observing a decrease of local recurrence following BCS. A multimodal approach in the management of breast cancer, including improved surgical techniques to assess negative margins, developed chemotherapy, and new targeted therapies, made this possible. The attitude of clinicians has evolved so that margins are now considered in a wider context that includes also tumour features, patient characteristics, and systemic and local treatment received. 

Oncological safety is the priority in oncological surgery; however, an important secondary goal in BCS is a good aesthetic result because it improves the patient's satisfaction and quality of life. Although many elements can affect the final cosmetic outcome, such as adjuvant therapy, tumour location, and other patient-related factors, the percentage of breast volume excised remains the most important factor. The implementation of the 2018 NICE guidelines that recommended “no ink on tumour” as an acceptable margin for invasive carcinoma and DCIS could have potentially decreased the re-excision rates [14]. In this cohort, 53% of re-excisions would have been avoided.

Thanks to national screening programmes, a large number of breast tumours are diagnosed at an early or non-invasive stage, when they are not palpable and therefore need to be localised prior to surgery to facilitate a complete excision. Wire-guided localisation, introduced in the 1970s, is still the most common technique despite known drawbacks and technological progress. A metallic wire is inserted by radiologists just before surgery - this requires co-ordination between radiology and surgery departments, which often results in a challenge for the services scheduling. In addition, the wire is easy to dislodge and it is often a cause for pain and anxiety. For these reasons, wireless techniques have been developed. The longest-established alternative to wire localisation up to now is radioactive seed localisation (RSL) [15]; however, this is not free of disadvantages, mainly due to the requirements for the handling of radioactive material. The limitations of wire-guided localisation and RSL have led to the development of some wireless radiation-free localisation techniques based on radio frequency and magnetometry [16,17].

We have recently investigated the performance of radio frequency identification (RFID) tags and magnetic seeds. Both devices are single-use metal markers implanted in the tumour with a needle under local anaesthetic any time before surgery. The markers are placed under ultrasound or stereotactic X-ray guidance and are detected in the operating room with a surgical probe. From systematic reviews of the literature, both RFID tags and magnetic seeds seem to be valid alternatives to wires in terms of safety, successful localisation, and margin positivity, with the advantage of decoupling surgery and radiology scheduling [18-20]. Other aspects, such as device manageability, aesthetic outcomes, and, not least, the financial impact on healthcare economics, should be assessed in future research to guide the choice of an alternative option to move from wires.

## Conclusions

DCIS at the resection margin, pure or combined with IDC, was the most significant factor associated with a re-excision in our cohort of patients.

The correlation between imaging size and actual histopathological size showed a statistically significant discrepancy in this cohort. Mammography provided the best correlation, followed by US scan and, lastly, MRI.
